# Promoting the use of elective single embryo transfer in clinical practice

**DOI:** 10.1186/s40738-016-0024-7

**Published:** 2016-08-15

**Authors:** Tamara Tobias, Fady I. Sharara, Jason M. Franasiak, Patrick W. Heiser, Emily Pinckney-Clark

**Affiliations:** 1 Seattle Reproductive Medicine, 1505 Westlake Ave North, Suite 400, Seattle, WA 98109 USA; 2 Virginia Center for Reproductive Medicine, 11150 Sunset Hills Rd, Suite #100, Reston, VA 20190 USA; 3 Department of Obstetrics and Gynecology, George Washington University, 2150 Pennsylvania Ave NW, Suite 6A 4169, Washington, DC 20037 USA; 4 Division of Reproductive Endocrinology, Department of Obstetrics, Gynecology and Reproductive Sciences, Robert Wood Johnson Medical School, Rutgers University, 125 Paterson St, New Brunswick, NJ 08901 USA; 5 Reproductive Medicine Associates of New Jersey, 140 Allen Road, Basking Ridge, NJ 07920 USA; 6 Ferring Pharmaceuticals, Inc., 100 Interpace Parkway, Parsippany, NJ 07054 USA

**Keywords:** Elective single embryo transfer, Assisted reproductive technologies, Patient education, Comprehensive chromosomal screening, Multiple births

## Abstract

**Background:**

The transfer of multiple embryos after in vitro fertilization (IVF) increases the risk of twins and higher-order births. Multiple births are associated with significant health risks and maternal and neonatal complications, as well as physical, emotional, and financial stresses that can strain families and increase the incidence of depression and anxiety disorders in parents. Elective single embryo transfer (eSET) is among the most effective methods to reduce the risk of multiple births with IVF.

**Main body:**

Current societal guidelines recommend eSET for patients <35 years of age with a good prognosis, yet even this approach is not widely applied. Many patients and clinicians have been reluctant to adopt eSET due to studies reporting higher live birth rates with the transfer of two or more embryos rather than eSET. Additional barriers to eSET include risk of treatment dropout after embryo transfer failure, patient preference for twins, a lack of knowledge about the risks and complications associated with multiple births, and the high costs of multiple IVF cycles. This review provides a comprehensive summary of strategies to increase the rate of eSET, including personalized counseling, access to educational information regarding the risks of multiple pregnancies and births, financial incentives, and tools to help predict the chances of IVF success. The use of comprehensive chromosomal screening to improve embryo selection has been shown to improve eSET outcomes and may increase acceptance of eSET.

**Conclusions:**

eSET is an effective method for reducing multiple pregnancies resulting from IVF. Although several factors may impede the adoption of eSET, there are a number of strategies and tools that may encourage the more widespread adoption of eSET in clinical practice.

## Background

Elective single embryo transfer (eSET) is the transfer of a single cleavage- or blastocyst-stage embryo after in vitro fertilization (IVF) or intracytoplasmic sperm injection (ICSI), despite the availability of more than one good-quality embryo [1]. Transfer of multiple embryos incurs an increased risk of twins or higher-order births, which can be associated with a variety of maternal and neonatal risks [2–4]. eSET is among the most effective methods to reduce the risk of multiple births with IVF [1]. The American Society for Reproductive Medicine (ASRM) and Society for Assisted Reproductive Technology (SART) have developed guidelines to help assist patients and clinicians in determining appropriate numbers of embryos to transfer and identifying those patients most appropriate for eSET [2, 4]. The guidelines currently recommend eSET for most patients aged <35 years with a good prognosis (Table 1) [2]. Although the chance of successful delivery declines with increasing age, patients of advanced age are still at risk for multiples and should be considered for eSET if they have top-quality embryos [2]; however, patients aged ≥35 years are not routinely offered SET [1, 5].

**Table 1:** ASRM/SART recommended criteria for eSET [1]

• Women aged <35 years − Women aged 35–40 years should also consider eSET if they have top-quality blastocyst-stage embryos available for transfer	
• More than one top-quality embryo available for transfer − Blastocyst-stage embryos are preferred	
• First or second IVF cycle	
• Previously successful IVF cycle	
• Recipients of embryos from donated eggs	

ASRM, American Society for Reproductive Medicine; SART, Society for Assisted Reproductive Technology; eSET, elective single embryo transfer; IVF, in vitro fertilization

Although SET rates have been increasing in the United States, they still lag significantly behind the rest of the world [1, 6]. In Europe, 27.5 % of all transfers in 2011 were SET [7], compared with rates in the United States of 17 % in 2011 and 24 % in 2013 [5, 8]. SET is mandated by the government in many European countries, and these policies have been shown to dramatically reduce the number of twin and higher-order births [1, 7, 9]. Implementation of a national SET policy in Sweden reduced the rate of twin births with IVF by 17 % without compromising outcomes (pregnancy rates were 33 % before and 37 % after SET legislation) [9]. Results from a survey of 170 fertility clinics in the United States showed that the majority (94 %) of respondents reported adhering to the ASRM transfer guidelines. However, 35 % also reported that they routinely transfer two embryos in patients <35 years of age with a good prognosis [10], a surprising disconnect. Moreover, half of the clinics indicated that they deviate from recommended guidelines in response to patient preferences. In 2013, the mean number of embryos transferred in fresh cycles in the United States was 1.8 for women <35 years of age and 1.9 for women 35 to 37 years of age, implying most centers still transfer two embryos in good-prognosis patients [5].

A number of factors are thought to impede the widespread uptake of eSET in the United States, including a perceived lower overall success rate when compared with double embryo transfer (DET); lack of patient education regarding the risks associated with multiples; and high dropout rate following failed cycles due to financial, emotional, and/or physical burden. Improving patient education and support is essential to encouraging the adoption of eSET, and predictive models and enhanced embryo selection can help to empower patients to choose eSET while maintaining high success rates. This review summarizes the benefits of eSET, obstacles to the adoption of eSET, and strategies to increase eSET rates to achieve the optimal outcome of infertility treatment—a healthy singleton birth.

### Outcomes with single versus multiple embryo transfer

**Fig. 1:**
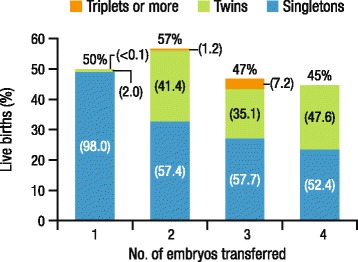
Live birth rates with SET and multiple embryo transfer [5]. Percentages of live births that were singletons, twins, and triplets or higher-order births are shown in parentheses. Percentages may not total 100.0 % due to rounding. In rare cases, a single embryo may divide and produce twins. For this reason, a small percentage of twins resulted from SET, and a small percentage of triplets resulted from DET. SET, single embryo transfer; DET, double embryo transfer

In the United States, the twin birth rate has increased by more than 75 % since 1980, mainly due to the use of assisted reproductive technology (ART) [3, 4]. The incidence of twin births is more than 20 times greater with pregnancies resulting from ART compared with naturally conceived pregnancies [4]. When comparing live birth rates for a single cycle with SET versus DET, the overall birth rate is higher with DET (a difference of 7 %); however, the proportion of multiple births is also substantially higher with DET (Fig. 1) [5, 11]. Results from a prospective, randomized trial in women aged <38 years with a good prognosis demonstrated that cumulative live birth rates with eSET followed by transfer of a single frozen embryo (45 %) were similar to those seen with a single DET cycle (42 %), whereas the rates of multiple births were 0 versus 28 %, respectively [12]. These findings are consistent with other studies [13–17] and suggest that initial eSET plus subsequent frozen eSET is as effective as DET while preventing the risks associated with multiple pregnancy.

### Impact of singleton versus multiple births

While medical advances have improved the outcomes of multiple births, these births are still associated with significant complications and health risks for both the mother and infants [3, 4]. Multiple birth parents also face increased financial and psychosocial stresses that can persist long after the newborn stage [3]. The medical, financial, and psychological effects of multiple pregnancy and birth on the mother and family are summarized in Table 2 and described below.

**Table 2:** Summary of the impacts of multiple pregnancy [3, 4, 18, 19, 21–26]

Maternal health	Infant health	Psychosocial effects on the family
• Pre-eclampsia • Gestational diabetes • Placental previa • Placental abruption • Preterm premature rupture of the membranes • Cesarean delivery • Postpartum hemorrhage • Death	• Placental problems − Premature aging − Twin-to-twin transfusion syndrome • Spontaneous abortion • Intrauterine growth restriction • Preterm (<37 weeks), very preterm (<32 weeks), and extreme preterm (<28 weeks) birth • Perinatal and infant mortality • Low (<2500 g) and very low birth weight (<1500 g) • Intraventricular hemorrhage • Periventricular leukomalacia • Respiratory distress syndrome • Bronchopulmonary dysplasia • Hypoxic-ischemic encephalopathy • Necrotizing enterocolitis • Sepsis • Jaundice • Retinopathy of prematurity • Cerebral palsy • Neural tube defects, heart malformations, and other birth defects • Developmental delays	• Postpartum depression (mother and father) • Relationship stress • Financial stress − Obstetric costs and neonatal intensive care admission − Costs for caring for multiple children throughout childhood

#### Maternal and infant health risks

A retrospective registry study comparing the outcomes of women undergoing two singleton pregnancies after IVF (*n* = 921) with those of women undergoing one twin pregnancy after IVF (*n* = 991) showed that neonatal and maternal outcomes were dramatically better in those undergoing two singleton pregnancies [18]. Indeed, multiple gestations increase the risk for nearly every recognized obstetric complication. Some of the significant maternal complications of multiple pregnancy include preeclampsia, gestational diabetes, placenta previa, placental abruption, and requirement for cesarean delivery [4, 18]. Twins and higher-order multiples are at greater risk for fetal growth restriction, preterm delivery, and low birth weight [4, 18, 19]. In 2013, >50 % of twins and >90 % of triplets in the United States were born preterm and/or had a low birth weight, compared with <10 % of singletons [20]. Preterm birth is associated with an increased risk of severe complications, including intraventricular hemorrhage, respiratory distress syndrome, necrotizing enterocolitis, sepsis, jaundice, and neonatal mortality [4, 18, 19]. Multiple births are also associated with an increased risk of longer-term complications, such as congenital heart defects, cerebral palsy, developmental delays, and learning disabilities [18, 21].

#### Financial considerations

The adverse medical outcomes associated with multiple births translate into significantly increased healthcare costs, particularly during infancy [22]. Estimated infant and maternal healthcare costs of twin pregnancies are three to five times higher than those of singleton pregnancies, while higher-order multiples cost approximately 20 times more than singletons [22, 23]. The increased healthcare costs of multiple births are due, in part, to complications during labor and delivery, requirement for cesarean delivery, longer duration of hospital stay, and increased admission to the neonatal intensive care unit (NICU) [23]. The increased need for/duration of bed rest during multiple pregnancies may also result in temporary loss of income [3], and these data are often not included in cost analyses of multiple gestations. Additionally, multiple births are associated with a variety of increased non-healthcare costs, including childcare for multiple children (or loss of income if a parent decides to stay at home), and additional costs for food, clothing, diapers, car seats, furniture/housing, schooling, and other essentials throughout childhood [3].

#### Psychosocial impacts

Physical, emotional, and financial stresses can increase the incidence of depression and anxiety disorders in parents rearing multiples [3, 24]. A survey of mothers raising children conceived through ART found that multiple births were associated with decreased quality of life and increased stress, depression, and social stigma [24]. Postpartum depression is also more common in parents of multiples and may be long term [3]. Studies have additionally reported that parents of multiples are more likely to experience decreased marital satisfaction [24] and to divorce or become separated compared with parents of singletons [25, 26]. A retrospective study in the United Kingdom also found that families with twins or triplets were more likely to be financially worse off after the births, to experience difficult or very difficult financial stress, to not have the mother return to work within 9 months after the birth, and to have children with delayed or very delayed school readiness [26].

### Barriers to eSET

Patient requests for multiple embryo transfer may represent the greatest challenge to the adoption of eSET as a standard of care. Multiple factors impact patients’ embryo transfer decision, including nulliparity, family income, level of knowledge regarding the risks of multiples, patient age, duration of infertility, and the desire to limit the physical and psychological stress of multiple IVF cycles [27–29].

Many patients express a preference for siblings, or even twins, and thus may be more willing to accept the risks associated with multiple pregnancy [28, 29]. A survey of 449 infertile women found that one in five listed multiple birth as their most desired outcome [28]. Avoidance of multiple births was indicated as less important than treatment efficacy, safety, affordability, and time to conception; patients who desired multiple births also demonstrated a greater lack of knowledge regarding the potential risks and complications associated with multiple births [28]. Thus, patients may truly desire a live birth and be less concerned, given lack of knowledge, how that perceived optimal outcome is achieved.

**Fig. 2:**
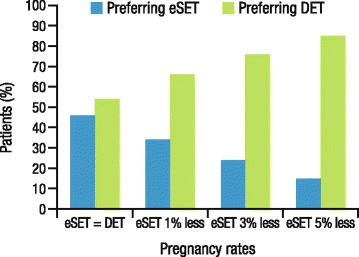
Patient preferences for eSET or DET in scenarios with varying pregnancy rates [32]. eSET, elective single embryo transfer; DET, double embryo transfer

For many infertile patients, the desire to become pregnant may outweigh concerns regarding the adverse outcomes of multiple births and the realities of raising multiple children, particularly given the increasing societal acceptance of multiples and their generally positive portrayal in the media [27, 30]. Women may be concerned that increasing age will limit their opportunities to have children. A study of 79 women undergoing IVF found that age was significantly correlated with embryo transfer preferences, with older women generally preferring to transfer multiple embryos [27]. A separate study found that patients with longer duration of infertility (≥2 years) were more likely to desire multiple births [28]. Infertile couples may want to maximize their chances for a successful outcome and consequently may be unwilling to accept a lower pregnancy rate to prevent a twin pregnancy [31]. Results from a study involving 244 women assessing the attitudes toward eSET versus DET in scenarios with various pregnancy rates demonstrated that when IVF/ICSI pregnancy rates were lowered by just 1–5 % after eSET, the proportion of patients preferring eSET decreased; in the scenario where eSET was as effective as DET, more than 50 % of patients still preferred DET over eSET (Fig. 2) [32]. All scenarios described a twin pregnancy rate of 25 % after DET versus 1 % after eSET. Additionally, patients who desire more than one child may want to attempt to complete their family in as few cycles as possible to avoid the emotional and psychological stress of further treatment [29].

Additional challenges to adopting eSET are the high out-of-pocket costs associated with IVF and limited insurance coverage for most patients in the United States [31]. A recent Canadian study found that just 1 year of universal IVF coverage increased eSET rates by 30 % and reduced multiple pregnancy by 23 % [33]. Similarly, implementation of an Australian healthcare program providing public funding for partial reimbursement of ART (including unlimited IVF cycles) resulted in an increase in eSET cycles from 29.5 to 68 % over a 6-year period [34]. In the United States, insurance coverage for IVF is also associated with fewer embryos transferred and a lower rate of multiple births; however, few states currently offer coverage for IVF [28, 35, 36].

### Strategies for increasing the eSET rate

**Fig. 3:**
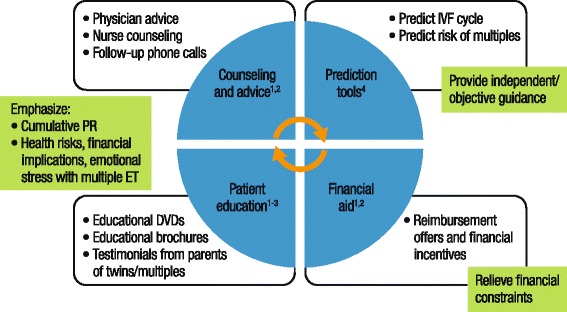
Strategies to increase acceptance of eSET [37–40]. eSET, elective single embryo transfer. IVF, in vitro fertilization; PR, pregnancy rate; ET, embryo transfer

Increasing eSET rates will have a large impact on reducing multiple births and should be encouraged early and consistently during treatment in good-prognosis patients [31]. It should be stressed that the goal of infertility treatment should be the delivery of a healthy single baby, with fewer twin and higher-order births. A multi-faceted approach incorporating patient education and counseling, reimbursement offers or other financial incentives, and IVF success prediction tools can be used to improve eSET rates in clinical practice (Fig. 3) [37–40].

#### Patient education and decision aids

Educational materials reviewing eSET and the risks and complications associated with multiple births can help patients make an informed decision on the number of embryos to transfer. Decision aids come in many forms, including written materials (e.g., fact sheets and brochures), websites, videos, interactive tools, and patient testimonials [38]. A randomized study in 222 infertile couples reported that the majority of patients appreciated the use of a decision aid and felt it helped them to decide how many embryos to transfer [38]. A New Zealand study showed that the rate of eSET tripled 1 year after the initiation of a patient education program providing materials that outlined the benefits of eSET and risks associated with DET [41]. A separate study evaluated the impact of an educational DVD or brochure on eSET rate and found that, after receiving the education materials, couples preferred eSET over DET, even among those who had previously preferred DET [39].

#### Counseling and advice from physicians and nurses

It is important for the entire team (physicians, nurses, embryologists, and other clinical staff) to be aligned with respect to eSET recommendations so that a clear, consistent message is provided to all patients. Nurses should objectively discuss the content of decision aids, clarify questions, and enable couples to make an informed decision [38]. A randomized study in 222 infertile couples showed that the physician’s advice and counseling by an IVF nurse were among the most influential factors driving a patient’s decision of how many embryos to transfer [38]. Investigators felt nurse counseling was important because nurses provided more individualized support for their patient’s particular physical, emotional, and social situation [38]. Nurses, in particular, can provide patients with tools to cope with the emotional burden of multiple ART cycles [42]. Approximately 70 % of patients also appreciated a follow-up phone call just prior to oocyte retrieval to discuss any relevant questions that might have arisen during IVF treatment.

#### Financial incentives

In the United States, insurance coverage for ART is mandated in only 15 states [36]. Reimbursement offers and financial incentives provided by fertility clinics or insurance companies may thus help relieve financial constraints that limit the number of cycles a patient can undergo and thereby influence a patient’s decision on how many embryos to transfer. Data from an ongoing pilot study evaluating the use of financial incentives (>$5000 in savings) to increase eSET rates in the United States showed that the majority of patients (60 %) agreed to eSET; the 40 % of couples who declined eSET did so because they desired twins [43]. Of note, the clinical and ongoing pregnancy rates were similar among patients in the eSET and DET groups; however, the rates of twin births were 2 and 30 %, respectively.

Several countries have implemented public funding or insurance programs that provide full or partial reimbursement of ART costs, sometimes contingent upon the transfer of a single or limited number of embryos. These programs have been associated with increases in the utilization of ART, live births following ART treatments, and the proportion of eSET or SET cycles, and a decrease in the rate of multiple births [34, 44–46]. These trends are observed regardless of whether SET is mandated or just encouraged.

#### Prediction tools

Information from online prediction tools that estimate the chances of IVF success based on individual patient characteristics can be used to help counsel patients [47–49]. Currently, patients can access several commercially available online prediction tools or a free online IVF predictor tool developed by SART. These tools incorporate a number of prognostic factors, such as a patient’s age, demographic information, health and medical history, and IVF response data, into a prediction model without considering data about embryo development, stage, and quality [40, 47, 49]. Prediction tools can be up to 1000 times more accurate in predicting a patient’s chance of successful outcome than simple age-based estimates [48], while also predicting the likelihood of multiple births. Information from these tools can complement personalized counseling in helping patients make decisions about whether and when to start IVF/ICSI procedures versus alternatives, such as intrauterine insemination or the use of donor eggs. Prediction tools can also help infertility center staff identify patients at greater risk for multiple births (e.g., probability >25 %) who should receive more extensive eSET counseling to minimize the risk of multiple gestation and associated complications.

#### Genetic screening

Preimplantation genetic screening (PGS), including comprehensive chromosomal screening (CCS) technologies, allows clinicians to assess embryos for aneuploidy (i.e., an abnormal number of chromosomes) prior to transfer [50]. Aneuploidy accounts for the majority (~70 %) of miscarriages in both natural and ART-conceived pregnancies [51]. Most patients undergoing IVF have at least one or two embryos available for biopsy, which involves removing a small number of cells for genetic testing. In a retrospective analysis of more than 15,000 embryos, the incidence of a patient having no normal (euploid) embryos was low for patients <40 years of age [50]. Trophectoderm biopsy at the blastocyst stage (Day 5 and/or 6) is currently the preferred method for screening [52], as it has, to date, not been shown to negatively impact implantation and pregnancy outcomes [50, 53]. The development of 24-chromosome CCS techniques, which currently include array comparative genomic hybridization (aCGH), quantitative real-time polymerase chain reaction (qPCR), single nucleotide polymorphism (SNP) array, and next-generation sequencing (NGS), has greatly improved the ability to detect aneuploid embryos [54]. In contrast with older methods (i.e., fluorescence in situ hybridization), these techniques allow for the analysis of all 24 chromosomes [54]. In a recent global survey of 386 IVF clinics where 342,000 cycles have been performed, aCGH was indicated as the preferred PGS method by 59 % of clinics, and NGS was indicated as the preferred method by 16 % [52].

**Fig. 4:**
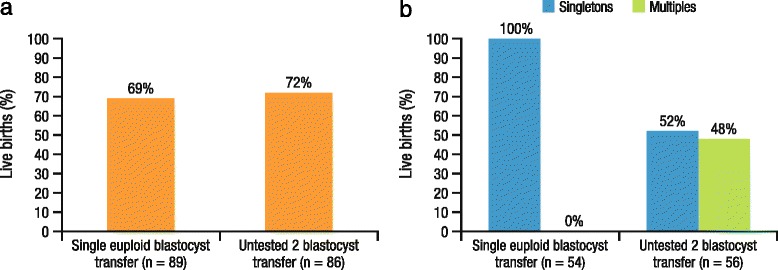
Impact of CCS and eSET on (**a**) delivery rates and (**b**) multiple births in patients undergoing IVF [59]. CCS, comprehensive chromosomal screening; eSET, elective single embryo transfer; IVF, in vitro fertilization

CCS is highly predictive of the reproductive potential of human embryos. Data from two prospective studies demonstrated that 96 % of embryos predicted to be aneuploid by CCS failed to sustain implantation compared with 41 % of embryos predicted to be euploid [55]. Recent meta-analyses also found that CCS increased implantation and pregnancy rates [56, 57]. Furthermore, use of CCS changes the selection of embryos for transfer compared with traditional morphology criteria, leading to the observed improvement in pregnancy and delivery outcomes [57, 58]. A separate randomized control study in 175 patients who were <43 years of age and undergoing IVF demonstrated that delivery rates were similar in patients receiving eSET with one euploid blastocyst versus those receiving DET with untested blastocysts (69 vs 72 %), with elimination of multiple pregnancies in the eSET group (Fig. 4) [59]. Euploid eSET was associated with improved health outcomes, including lower risk of preterm delivery and NICU admission, and reduced costs compared with untested DET [59]. The use of CCS may also improve SET outcomes in patients of advanced maternal age. Initial findings from a study in patients aged >35 years showed that pregnancy rates were higher in patients who underwent eSET with a frozen euploid embryo compared with those who underwent eSET with a fresh untested embryo (61 vs 41 %, respectively) [51]. It should be noted that both fresh and frozen embryo transfers can be used in IVF clinics following CCS [52].

Given that embryo selection based on CCS results in improved outcomes, patients may be more willing to choose eSET if CCS options are utilized to guide embryo selection. The previously mentioned global survey indicated that 43 % of surveyed IVF clinics currently use PGS in less than 10 % of cycles, with another 20 % of clinics using PGS in just 10–20 % of cycles, although it is suggested that a much higher proportion of patients are considered eligible for PGS [52]. The low rate of PGS use may be due in part to the requirement of patients to cover the costs out of pocket [52], although these costs are more than offset by lower hospital costs (through 28 days post-delivery) with euploid CCS eSET versus untested DET, due to the elimination of multiple births [60]. Additionally, as the technology evolves, the costs for testing continue to decrease.

### Mandatory SET policies and regulations

Several countries have developed policies and regulations concerning the number of embryos transferred during IVF cycles. In some countries, such as Sweden, concerns over the high rate of multiple births led to government legislation limiting most patients to SET, with the transfer of two or three embryos permitted only in certain subpopulations [61]. Several other countries have regulations on the number of transferred embryos that are tied to insurance and public funding programs that provide comprehensive or partial coverage for ART. However, patients are not forced to adhere to these regulations if they prefer to pay for ART themselves [34, 44–46]. In Finland, there is no formal legislation or requirement for SET, but IVF clinics together elected to move towards an SET policy for most patients [62]. Regardless of the specific type of policy set forth, retrospective studies comparing IVF outcomes from before and after implementation of these initiatives consistently demonstrate a sizeable decrease in the rates of multiple pregnancies and multiple live births (e.g., from 25 to 5 %) while maintaining steady cumulative clinical pregnancy and live birth rates. A Belgian study further showed that the cumulative delivery rate for patients’ first two cycles was lower after introduction of a reimbursement/SET policy, but that it had no statistically significant impact on the 6-cycle cumulative delivery rate (65 % before vs 60 % after) [45]. Initiation of the health program in Quebec was also associated with a decrease of 35.5 % in premature live births and 37 % in the requirement for NICU admission [44].

Currently there are no embryo transfer regulations in the United States, although ASRM and SART have put forth guidelines to assist centers in determining the appropriate number of embryos for transfer in their patients [2, 4]. In some instances, individual IVF centers have chosen to develop mandatory SET policies for all or certain subsets of their patients. If such a policy is put in place, the center should ensure that it is clearly described to all patients on their first visit and again throughout the IVF cycle (e.g., prior to oocyte retrieval and transfer) to help set and reinforce expectations.

One US clinic developed a mandatory SET policy for all women <38 years of age without a history of failed fresh cycle at their center, with ≥7 zygotes (2-pronuclei stage) for culture, and ≥1 good- or excellent-quality blastocyst available for transfer [63]. A retrospective analysis of all women <38 years of age undergoing a fresh transfer at their center found that, in the 5 years following implementation of the mandatory SET policy, the overall live birth rate significantly improved from 51 to 56 % (*P* = 0.026) and the multiple birth rate decreased from 35 to 17.5 % (*P* < 0.0001). Among the women who underwent mandatory SET, the live birth rate was 66 % (cumulative rate of 84 %) and the multiple birth rate was 3.4 %. Of note, implementation of the mandatory SET policy did not affect overall clinical volume (2412 cycles in the prior 5 years; 2389 cycles in the subsequent 5 years) [63]. A survey performed by the same US center found that 94 % of all patients supported the mandatory SET policy (including 69 % who strongly supported it) [64]. Support for the policy did not vary by the number of embryos transferred, but was stronger among patients who felt they had the right amount of input into their IVF treatment and embryo transfer decision, expressed concerns about multiples, had extra embryos for cryopreservation, and/or had a shorter duration of infertility.

## Conclusions

eSET is an effective method for reducing multiple pregnancies resulting from IVF and should be consistently encouraged for the majority of patients to improve the likelihood of delivering a healthy baby. There are many factors that impede the adoption of eSET. Overall success rates are lower per fresh cycle when compared with the transfer of two or more embryos [8, 11, 13]. Patients may not be willing to risk a failed cycle given the financial, emotional, and physical burden associated with IVF. Furthermore, many patients desire twins and do not fully consider the risks associated with twin pregnancies and births [28, 29]. However, providing patients with personalized counseling, access to educational information regarding the risks of DET and multiple births, financial incentives, tools to help predict the chances of IVF success, and technologies to select high-quality embryos for transfer should help to increase the use of eSET in clinical practice.

## Abbreviations

aCGH, array comparative genomic hybridization; ART, assisted reproductive technology; ASRM, American Society for Reproductive Medicine; CCS, comprehensive chromosomal screening; DET, double embryo transfer; eSET, elective single embryo transfer; ICSI, intracytoplasmic sperm injection; IVF, in vitro fertilization; NGS, next-generation sequencing; NICU, neonatal intensive care unit; PGS, preimplantation genetic screening; qPCR, quantitative real-time polymerase chain reaction; SART, Society for Assisted Reproductive Technology; SET, single embryo transfer; SNP, single nucleotide polymorphism

## References

[1] American Society for Reproductive Medicine (2012). Elective single-embryo transfer. Fertil Steril.

[2] Practice Committee of the American Society for Reproductive Medicine, Practice Committee of the Society for Assisted Reproductive Technology (2013). Criteria for number of embryos to transfer: a committee opinion. Fertil Steril.

[3] American Society for Reproductive Medicine (2012). Multiple Pregnancy and Birth: Twins, Triplets, and High-order Multiples: A Guide for Patients.

[4] American Society for Reproductive Medicine (2012). Multiple gestation associated with infertility therapy: an American Society for Reproductive Medicine Practice Committee opinion. Fertil Steril.

[5] Centers for Disease Control and Prevention (CDC), American Society for Reproductive Medicine, Society for Assisted Reproductive Technology. 2013 Assisted Reproductive Technology: National Summary Report. Atlanta: US Department of Health and Human Services; 2015.

[6] Maheshwari A, Griffiths S, Bhattacharya S (2011). Global variations in the uptake of single embryo transfer. Hum Reprod Update.

[7] Kupka MS, D'Hooghe T, Ferraretti AP, De MJ, Erb K, Castilla JA (2016). Assisted reproductive technology in Europe, 2011: results generated from European registers by ESHREdagger. Hum Reprod.

[8] Centers for Disease Control and Prevention (CDC), American Society for Reproductive Medicine, Society for Assisted Reproductive Technology (2013). 2011 Assisted Reproductive Technology: National Summary Report.

[9] Saldeen P, Sundstrom P (2005). Would legislation imposing single embryo transfer be a feasible way to reduce the rate of multiple pregnancies after IVF treatment?. Hum Reprod.

[10] Jungheim ES, Ryan GL, Levens ED, Cunningham AF, Macones GA, Carson KR (2010). Embryo transfer practices in the United States: a survey of clinics registered with the Society for Assisted Reproductive Technology. Fertil Steril.

[11] Pandian Z, Bhattacharya S, Ozturk O, Serour G, Templeton A (2009). Number of embryos for transfer following in-vitro fertilisation or intra-cytoplasmic sperm injection. Cochrane Database Syst Rev.

[12] Lopez-Regalado ML, Clavero A, Gonzalvo MC, Serrano M, Martinez L, Mozas J (2014). Randomised clinical trial comparing elective single-embryo transfer followed by single-embryo cryotransfer versus double embryo transfer. Eur J Obstet Gynecol Reprod Biol.

[13] Clua E, Tur R, Coroleu B, Rodriguez I, Boada M, Gomez MJ (2015). Is it justified to transfer two embryos in oocyte donation? A pilot randomized clinical trial. Reprod Biomed Online.

[14] Thurin A, Hausken J, Hillensjo T, Jablonowska B, Pinborg A, Strandell A (2004). Elective single-embryo transfer versus double-embryo transfer in in vitro fertilization. N Engl J Med.

[15] Criniti A, Thyer A, Chow G, Lin P, Klein N, Soules M (2005). Elective single blastocyst transfer reduces twin rates without compromising pregnancy rates. Fertil Steril.

[16] Henman M, Catt JW, Wood T, Bowman MC, de Boer KA, Jansen RP (2005). Elective transfer of single fresh blastocysts and later transfer of cryostored blastocysts reduces the twin pregnancy rate and can improve the in vitro fertilization live birth rate in younger women. Fertil Steril.

[17] Le LD, Griveau JF, Laurent MC, Gueho A, Veron E, Morcel K (2006). Contribution of embryo cryopreservation to elective single embryo transfer in IVF-ICSI. Reprod Biomed Online.

[18] Sazonova A, Kallen K, Thurin-Kjellberg A, Wennerholm UB, Bergh C (2013). Neonatal and maternal outcomes comparing women undergoing two in vitro fertilization (IVF) singleton pregnancies and women undergoing one IVF twin pregnancy. Fertil Steril.

[19] Pinborg A, Loft A, Nyboe AA (2004). Neonatal outcome in a Danish national cohort of 8602 children born after in vitro fertilization or intracytoplasmic sperm injection: the role of twin pregnancy. Acta Obstet Gynecol Scand.

[20] Martin JA, Hamilton BE, Osterman MJ, Curtin SC, Matthews TJ (2015). Births: final data for 2013. Natl Vital Stat Rep.

[21] Moore AM, O'Brien K (2006). Follow-up issues with multiples. Paediatr Child Health.

[22] Chambers GM, Ledger W (2014). The economic implications of multiple pregnancy following ART. Semin Fetal Neonatal Med.

[23] Lemos EV, Zhang D, Van Voorhis BJ, Hu XH (2013). Healthcare expenses associated with multiple vs singleton pregnancies in the United States. Am J Obstet Gynecol.

[24] Ellison MA, Hotamisligil S, Lee H, Rich-Edwards JW, Pang SC, Hall JE (2005). Psychosocial risks associated with multiple births resulting from assisted reproduction. Fertil Steril.

[25] Jena AB, Goldman DP, Joyce G (2011). Association between the birth of twins and parental divorce. Obstet Gynecol.

[26] McKay S. The Effects of Twins and Multiple Births on Families and Their Living Standards. Aldershot, United Kingdom: Twins & Multiple Births Association (TAMBA); 2010.

[27] Newton CR, McBride J, Feyles V, Tekpetey F, Power S (2007). Factors affecting patients' attitudes toward single- and multiple-embryo transfer. Fertil Steril.

[28] Ryan GL, Zhang SH, Dokras A, Syrop CH, Van Voorhis BJ (2004). The desire of infertile patients for multiple births. Fertil Steril.

[29] Ismail L, Mittal M, Kalu E (2012). IVF twins: buy one get one free?. J Fam Plann Reprod Health Care.

[30] Letterie GS (2004). Multiple births: does the news media influence public perceptions?. Hum Reprod.

[31] Kissin DM, Kulkarni AD, Mneimneh A, Warner L, Boulet SL, Crawford S (2015). Embryo transfer practices and multiple births resulting from assisted reproductive technology: an opportunity for prevention. Fertil Steril.

[32] Twisk M, van der Veen F, Repping S, Heineman MJ, Korevaar JC, Bossuyt PM (2007). Preferences of subfertile women regarding elective single embryo transfer: additional in vitro fertilization cycles are acceptable, lower pregnancy rates are not. Fertil Steril.

[33] Velez MP, Connolly MP, Kadoch IJ, Phillips S, Bissonnette F (2014). Universal coverage of IVF pays off. Hum Reprod.

[34] Chambers GM, Illingworth PJ, Sullivan EA (2011). Assisted reproductive technology: public funding and the voluntary shift to single embryo transfer in Australia. Med J Aust.

[35] Jain T, Harlow BL, Hornstein MD (2002). Insurance coverage and outcomes of in vitro fertilization. N Engl J Med.

[36] National Conference of State Legislatures. State laws related to insurance coverage for infertility treatment. http://www.ncsl.org/research/health/insurance-coverage-for-infertility-laws.aspx (2015). Accessed 25 Sept 2015.

[37] Van Peperstraten A, Nelen W, Grol R, Zielhuis G, Adang E, Stalmeier P (2010). The effect of a multifaceted empowerment strategy on decision making about the number of embryos transferred in in vitro fertilisation: randomised controlled trial. BMJ.

[38] Kreuwel IA, van Peperstraten AM, Hulscher ME, Kremer JA, Grol RP, Nelen WL (2013). Evaluation of an effective multifaceted implementation strategy for elective single-embryo transfer after in vitro fertilization. Hum Reprod.

[39] Hope N, Rombauts L (2010). Can an educational DVD improve the acceptability of elective single embryo transfer? A randomized controlled study. Fertil Steril.

[40] Lannon BM, Choi B, Hacker MR, Dodge LE, Malizia BA, Barrett CB (2012). Predicting personalized multiple birth risks after in vitro fertilization-double embryo transfer. Fertil Steril.

[41] Coetzee K, Stewart B, Peek J, Hutton JD (2007). Acceptance of single-embryo transfer by patients. Fertil Steril.

[42] Roca-de BM, Gutierrez-Maldonado J, Gris-Martinez JM (2011). Comparative study of the psychosocial risks associated with families with multiple births resulting from assisted reproductive technology (ART) and without ART. Fertil Steril.

[43] Sharara FI (2015). Despite significant financial incentives many couples still decline elective single embryo transfers (eSET). Fertil Steril.

[44] Shaulov T, Belisle S, Dahan MH (2015). Public health implications of a North American publicly funded in vitro fertilization program; lessons to learn. J Assist Reprod Genet.

[45] Peeraer K, Debrock S, Laenen A, De LP, Spiessens C, De ND (2014). The impact of legally restricted embryo transfer and reimbursement policy on cumulative delivery rate after treatment with assisted reproduction technology. Hum Reprod.

[46] Knez J, Kovacic B, Vlaisavljevic V (2013). Comparison of embryo transfer strategies and assisted reproduction outcome in Slovenian and cross-border patients. Reprod Biomed Online.

[47] Luke B, Brown MB, Wantman E, Stern JE, Baker VL, Widra E (2014). A prediction model for live birth and multiple births within the first three cycles of assisted reproductive technology. Fertil Steril.

[48] Choi B, Bosch E, Lannon BM, Leveille MC, Wong WH, Leader A (2013). Personalized prediction of first-cycle in vitro fertilization success. Fertil Steril.

[49] Banerjee P, Choi B, Shahine LK, Jun SH, O'Leary K, Lathi RB (2010). Deep phenotyping to predict live birth outcomes in in vitro fertilization. Proc Natl Acad Sci U S A.

[50] Franasiak JM, Forman EJ, Hong KH, Werner MD, Upham KM, Treff NR (2014). The nature of aneuploidy with increasing age of the female partner: a review of 15,169 consecutive trophectoderm biopsies evaluated with comprehensive chromosomal screening. Fertil Steril.

[51] Schoolcraft WB, Katz-Jaffe MG (2013). Comprehensive chromosome screening of trophectoderm with vitrification facilitates elective single-embryo transfer for infertile women with advanced maternal age. Fertil Steril.

[52] Wiessman A, Yaron Y, Fishel S. Preimplantation genetic screening (PGS): what is my opinion? [survey] IVF Worldwide. http://www.ivf-worldwide.com/survey/preimplantation-genetic-screening-pgs-what-is-my-opinion/results-preimplantation-genetic-screening-pgs-what-is-my-opinion.html. Accessed 21 Jan 2016.

[53] Scott RT, Upham KM, Forman EJ, Zhao T, Treff NR (2013). Cleavage-stage biopsy significantly impairs human embryonic implantation potential while blastocyst biopsy does not: a randomized and paired clinical trial. Fertil Steril.

[54] Wu MY, Chao KH, Chen CD, Chang LJ, Chen SU, Yang YS (2014). Current status of comprehensive chromosome screening for elective single-embryo transfer. Obstet Gynecol Int.

[55] Scott RT, Ferry K, Su J, Tao X, Scott K, Treff NR (2012). Comprehensive chromosome screening is highly predictive of the reproductive potential of human embryos: a prospective, blinded, nonselection study. Fertil Steril.

[56] Dahdouh EM, Balayla J, Garcia-Velasco JA (2015). Comprehensive chromosome screening improves embryo selection: a meta-analysis. Fertil Steril.

[57] Chen M, Wei S, Hu J, Quan S (2015). Can comprehensive chromosome screening technology improve IVF/ICSI outcomes? A meta-analysis. PLoS One.

[58] Scott RT, Upham KM, Forman EJ, Hong KH, Scott KL, Taylor D (2013). Blastocyst biopsy with comprehensive chromosome screening and fresh embryo transfer significantly increases in vitro fertilization implantation and delivery rates: a randomized controlled trial. Fertil Steril.

[59] Forman EJ, Hong KH, Franasiak JM, Scott RT (2014). Obstetrical and neonatal outcomes from the BEST trial: single embryo transfer with aneuploidy screening improves outcomes after in vitro fertilization without compromising delivery rates. Am J Obstet Gynecol.

[60] Forman J, Hong KH, Werner MD, Singer SA, Benson MR, Scott RT (2013). Reducing the burden of art care: Single blastocyst transfer after comprehensive chromosome screening (CCS) provides equivalent delivery rates, eliminates twins and lowers global health care costs. Fertil Steril.

[61] Karlstrom PO, Bergh C (2007). Reducing the number of embryos transferred in Sweden—impact on delivery and multiple birth rates. Hum Reprod.

[62] Hamberger L, Hardarson T, Nygren KG (2005). Avoidance of multiple pregnancy by use of single embryo transfer. Minerva Ginecol.

[63] Kresowik JD, Stegmann BJ, Sparks AE, Ryan GL, Van Voorhis BJ (2011). Five-years of a mandatory single-embryo transfer (mSET) policy dramatically reduces twinning rate without lowering pregnancy rates. Fertil Steril.

[64] Martini S, Van Voorhis BJ, Stegmann BJ, Sparks AE, Shochet T, Zimmerman MB (2011). In vitro fertilization patients support a single blastocyst transfer policy. Fertil Steril.

